# Evaluating the Efficacy of Infliximab in Inflammatory Bowel Disease: A Systematic Review of the Literature

**DOI:** 10.7759/cureus.65971

**Published:** 2024-08-01

**Authors:** Maria P Vallejo, Arturo P Jaramillo, Carlos Luis Guanín Cabrera, Maria G Cueva, Mario Navarro Grijalva, Xavier Grandes

**Affiliations:** 1 Internal Medicine, Universidad Católica de Santiago de Guayaquil, Guayaquil, ECU; 2 General Practice, Universidad Estatal de Guayaquil, Machala, ECU; 3 General Practice, Universidad Católica de Santiago de Guayaquil, Guayaquil, ECU; 4 Urology, Universidad Católica de Santiago de Guayaquil, Guayaquil, ECU; 5 Research, Universidad Católica de Santiago de Guayaquil, Guayaquil, ECU

**Keywords:** infliximab ct-p13, biological therapy, infliximab, infliximab biosimilar, inflammatory bowel disease

## Abstract

The introduction of steroid therapy in 1955 markedly decreased the mortality rate of severe ulcerative colitis (UC) from 24% in the placebo group to 7%, and it is currently less than 1% in specialist centers. Despite this advancement, the response of severe UC to steroids has stagnated over the past 50 years, with a high rate of colectomy persisting for severe to moderately severe cases. Infliximab (IFX) (Remicade, Centocor Inc., Malvern, PA, United States), an intravenously administered chimeric monoclonal immunoglobulin G1 antibody targeting tumor necrosis factor-alpha (TNF-α), has shown efficacy in numerous randomized controlled trials for treating moderate to severe and fistulizing Crohn's disease, particularly in patients unresponsive to conventional therapies. This led to its approval by the US Food and Drug Administration in 1998 for treating active and fistulizing Crohn's disease. Most clinical research on IFX has focused on Crohn's disease, which is characterized as a Th1-type condition driven by pro-inflammatory cytokines like TNF-α. Conversely, UC has traditionally been viewed as a Th2-type condition where TNF-α plays a lesser role. However, recent studies indicate that TNF-α might also contribute to the pathogenesis of UC. These findings highlight the necessity for larger randomized controlled trials to further investigate the benefits of therapies like IFX, with the ultimate goal of improving treatment outcomes and quality of life for patients with inflammatory bowel disease.

## Introduction and background

Inflammatory bowel disease (IBD), which encompasses Crohn’s disease (CD) and ulcerative colitis (UC), is marked by chronic, relapsing inflammation of the intestines. It poses a significant global health concern because of its increasing incidence. The prevailing understanding is that IBD arises from an abnormal and persistent immune response to gut microbes, driven by the individual's genetic susceptibility. Although the exact cause of IBD remains largely elusive, it is believed to result from a complex interplay between genetic, environmental, microbial factors, and immune responses [1,2,3,4]. The genetic study of gut inflammation, one of the four key components of IBD pathogenesis, has made significant advancements. Recent international collaborative studies have identified 163 gene loci associated with IBD susceptibility. Interestingly, the genetic predispositions for childhood-onset and adult-onset IBD appear to overlap, indicating similar genetic factors at play. Even though it's not clear what causes UC and CD, it is clear that immune systems become active in the inflamed mucosa, which contributes to the development of chronic diseases [4]. The mucosa produces more immunoglobulins, some of which target bacterial antigens, and disease exacerbations trigger complement activation. Serum factors and bacterial antigens influence the cytotoxicity of lymphocytes isolated from both peripheral blood and intestinal mucosa to colonic epithelial cells in vitro. Within the mucosa, there is an increased presence of T lymphocytes, although the helper-to-suppressor cell ratio remains unchanged phenotypically [5]. Research into immunoregulatory control has revealed potential alterations in the local immune response modulation, particularly during active disease phases. However, it remains unclear whether these changes are primary or secondary to inflammation [2,3]. We hypothesize that increased antigen absorption and enhanced antigen presentation to the immune system trigger many humoral and cellular immune responses to gut-associated antigens. This is due to the expression of Class II antigens by the inflamed epithelium and altered immunoregulatory control [3,5].

Using anti-tumor necrosis factor α (anti-TNF-α) agents, like infliximab (IFX) (Remicade, Centocor Inc., Malvern, PA, United States), has proven to be clinically effective in treating patients with CD. However, the extensive use of these agents has placed a significant financial strain on healthcare systems worldwide [1,2]. The significant price difference between biosimilar infliximab (CT-P13) and the original IFX for maintenance therapy highlights the financial consequences of this situation [3]. Biological agents possess the ability to initiate the production of antibodies that specifically target the administered drug. This can lead to a decrease in the level of the drug in the bloodstream [4]. In a significant number of patients, immunogenicity may result in primary nonresponse, heightened infusion reactions, and a decline in response over time. It is evident that there is a direct relationship between the original IFX and clinical outcomes, as higher serum levels are associated with positive results. Paul et al. did a study and found that keeping a delta IFX serum concentration above 0.5 g/mL at week eight was the only thing that was linked to endoscopic remission in people with inflammatory bowel disease (IBD). This finding had a likelihood ratio of 2.02 and a 95% confidence interval of 1.01 to 4.08, with a p-value of 0.048 [5]. In the same way, Papamichael et al. found that IFX levels in the blood (≥2.2, ≥9.7, and ≥9.8 μg/mL) were related to biochemical, endoscopic, and histologic remission, in that order [6,7].

Recent consensus statements have highlighted the significance of therapeutic drug monitoring for anti-TNF-α therapy. However, depending on the clinical outcomes under assessment, the ideal drug concentrations and anti-drug antibody (ADA) thresholds may vary [6,7]. In CD patients, we expect CT-P13 to exhibit comparable immunogenicity and drug concentration levels to the originator IFX [8,9]. IBD patients' ADA against the original drug IFX also hinders CT-P13's function, indicating a cross-reactivity and comparable immunogenicity between these two medications. It is clear that there is a relationship between exposure to CT-P13 during the induction period and improved clinical outcomes when serum IFX levels are higher. There is a correlation between the initial IFX trough levels at week two and the effectiveness of treatment at weeks 14 and 30 in patients with UC who received CT-P13 [10]. An indicator of steroid-free clinical remission and mucosal healing at week 14 was a cutoff value of 3.15 μg/mL [11].

However, studies comparing serum trough levels of CT-P13 with those of its originator have produced conflicting findings. The SECURE trial, a Phase 4 study across multiple centers, demonstrated that IBD patients who achieved clinical remission and switched to CT-P13 from the originator IFX experienced similar serum concentration and ADA levels [12,13]. On the other hand, a study that looked at old data with only a few participants found that the original IFX group and the CT-P13 group had different serum IFX trough levels at week 22 in IBD patients who were not switching groups. However, it's important to note that the study's design raises questions about the accuracy of these findings.

Research has demonstrated that using a combination of IFX and azathioprine is more successful in achieving clinical remission and endoscopic healing in patients who have not previously used azathioprine. This highlights the importance of early disease management and combining treatments to improve the chances of successful treatment. Nevertheless, after achieving remission, it is crucial to thoroughly consider the advantages and disadvantages of maintaining combination therapy with IFX and an immunosuppressant (such as thiopurines or methotrexate). There is a growing consensus that the risks of serious infections and lymphoproliferative disorders are higher with combination therapy compared to monotherapy [14]. It is important to be aware of these increased risks. Registry data indicate that reducing drug therapy could lower the chances of experiencing harmful drug-related side effects and potentially save money [14]. However, the potential for disease relapse when stopping either or both drugs remains uncertain, as does the increased risk of immunogenicity when discontinuing immunosuppressant therapy while continuing IFX. In a randomized trial, patients who stopped immunosuppressant therapy did not experience a higher rate of treatment failure compared to those who continued combination therapy [14]. The main objective of this systematic review is to comprehensively analyze recent studies on the efficacy of IFX in patients with IBD. This review aims to improve understanding of IFX treatment outcomes and provide valuable insights for effectively managing IBD through a critical evaluation of the latest research.

## Review

Methods

Review Records and Search for Studies

This systematic review followed the requirements of the Preferred Reporting Items for Systematic Reviews and Meta-Analyses (PRISMA) [15]. Independent researchers conducted exhaustive searches in Pubmed, Pubmed Central, and the Cochrane Library to choose articles. Table 1 provides details on the search methods used.

**Table 1:** Search strategy for databases

Search Strategy	Databases Used	Number of Papers Identified
Inflammatory Bowel Disease AND Infliximab AND Biological Therapy	Pubmed	1859
( "Infliximab/administration and dosage"[Majr] OR "Infliximab/analysis"[Majr] OR "Infliximab/drug effects"[Majr] OR "Infliximab/therapeutic use"[Majr] )	Pubmed Central (PMC)	423
"Inflammatory Bowel Disease [tw]" AND "Infliximab [tiab]" AND " Biological Therapy [all]"	Cochrane Library	330

Inclusion and Exclusion Criteria

Two independent authors used the Covidence program to filter search results from two databases using predefined inclusion and exclusion criteria, as shown in Table 2. 

**Table 2:** Inclusion and exclusion criteria

Inclusion	Exclusion
Free, full text about infliximab effectiveness	Articles that do not include biological therapeutics
Articles from the past 10 years	Articles from 2013 and below
English-language articles	Non-English studies
Prospective or retrospective studies	Case reports
Human trials	Animal trials

The implementation of the PICO format for the systematic review will be shown in Table 3.

**Table 3:** PICO format for systematic review PICO: population, intervention, comparison, outcome; IBD: inflammatory bowel disease; CD: Crohn's disease; UC: ulcerative colitis; TNF-α: tumor necrosis factor-alpha; IFX: infliximab

Population (P)	Intervention (I)	Comparison (C)	Outcome (O)
Patients diagnosed with IBD, specifically those with CD and UC	Treatment with IFX, a biologic therapy targeting TNF-α	Placebo treatment; Standard care or other therapeutic agents (e.g., corticosteroids, aminosalicylates, immunosuppressants)	Primary outcomes: Clinical remission, mucosal healing, and reduction in disease activity; Secondary outcomes: Quality of life, hospitalization rates, surgical intervention rates, and adverse effects associated with treatment

Data Extraction

An exhaustive investigation of the pertinent study yielded a number of significant findings. An example of this is the design of each study, which primarily focuses on the biological treatment using infliximab in patients who have inflammatory bowel disease (IBD).

Risk of Bias Assessment

We used the Cochrane risk of bias tool, designed for randomized controlled trials (RCTs), to assess potential biases in the selected studies for our inquiry. This was conducted to assess if the studies had any potential biases. This tool has gained considerable recognition for its efficacy in assessing the quality of research that relies on case series [16]. A thorough assessment of potential bias in each research piece was conducted by the reviewers, and any differences in their evaluations were resolved through meaningful discussions.

Results

**Figure 1:**
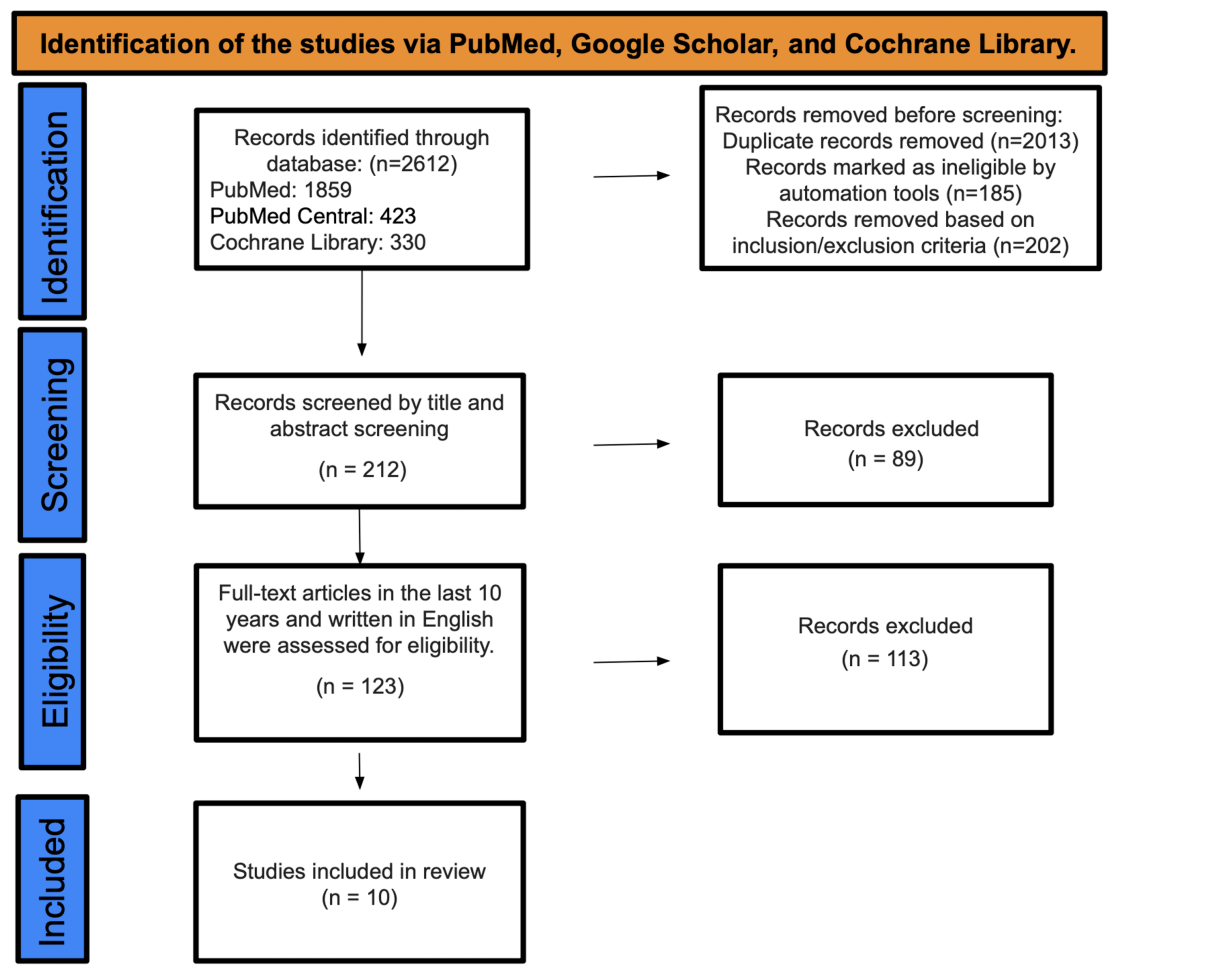
PRISMA diagram PRISMA: Preferred Reporting Items for Systematic Reviews and Meta-Analyses

After searching the PubMed, PMC, and Cochrane Library databases, a total of 2612 studies were discovered. A total of 202 studies were deemed ineligible due to inclusion and exclusion criteria, and 2013 duplicate studies were eliminated. A total of 212 studies underwent title and abstract screening, and 89 papers were discarded as they were not relevant to our study's purpose. Out of the initial pool of 123 papers, a thorough evaluation was conducted based on their English content and full-text availability over the past decade. As a result, 113 studies were excluded, leaving only 10 studies that met the criteria for the final data collection (Figure 1). 

A list of 20 potential articles on the effectiveness of IFX in treating IBD was created through a comprehensive literature search, which then informed the study's questionnaire. The Delphi technique was employed to reach a consensus among experts on the importance of various topics related to IFX in IBD patients. The consistency of responses across different rounds was analyzed using the chi-square test. The initial consensus percentages from Round 1 were considered the observed range, while those from Round 2 served as the expected range. A P-value of 0.0005 indicated a highly stable set of responses. Table 4 categorizes the identified needs/topics by discipline as presented to the expert panel.

**Table 4:** Questionnaire topics * Strikethrough: topics that remained excluded after the last Delphi round.

Discipline	Topics	Consensus%
Gastroenterology	Early infliximab trough levels predict the long-term efficacy of infliximab in a randomized controlled trial in patients with active Crohn’s disease comparing, CT-P13 and originator infliximab [13]	90.5
	Withdrawal of infliximab or concomitant immunosuppressant therapy in patients with Crohn's disease on combination therapy (SPARE): a multicentre, open-label, randomized controlled trial [14]	85.2
	VE303, a defined bacterial consortium, for prevention of recurrent Clostridioides difficile infection: a randomized clinical trial [17]	88.6
	Effect of therapeutic drug monitoring vs standard therapy during maintenance infliximab therapy on disease control in patients with immune-mediated inflammatory diseases: a randomized clinical trial [18]	76.5
	Effect of therapeutic drug monitoring vs standard therapy during infliximab induction on disease remission in patients with chronic immune-mediated inflammatory diseases: a randomized clinical trial [19]	72.5
Immunology	Infliximab, azathioprine, or combination therapy for Crohn's disease	59.9
	Randomized, double-blind, phase III study comparing the infliximab biosimilar, PF-06438179/GP1111, with reference infliximab: efficacy, safety, and immunogenicity from week 30 to week 54	62.1
	Influence of immunogenicity on the long-term efficacy of infliximab in Crohn's disease	51.3
Therapeutics	Infliximab concentration monitoring improves the control of disease activity in rheumatoid arthritis	65.1
	Infliximab maintenance therapy for fistulizing Crohn's disease	63.8
	Randomized controlled trial: subcutaneous vs intravenous infliximab CT-P13 maintenance in inflammatory bowel disease [20]	97.5
	Efficacy and safety of CT-P13 in inflammatory bowel disease after switching from originator infliximab: exploratory analyses from the NOR-SWITCH main and extension trials [21]	83.6
Pathology	Infliximab reduces the number of activated mucosal lymphocytes in patients with Crohn's disease	47.1
	Increasing infliximab dose based on symptoms, biomarkers, and serum drug concentrations does not increase clinical, endoscopic, and corticosteroid-free remission in patients with active luminal Crohn’s disease [22]	89.7
	UK Dermatology Clinical Trials Network's STOP GAP trial (a multicentre trial of prednisolone versus ciclosporin for pyoderma gangrenosum): protocol for a randomized controlled trial	59.9
Infectious disease	Infliximab for the treatment of fistulas in patients with Crohn's disease	51.2
	Maintenance infliximab does not result in increased abscess development in fistulizing Crohn's disease: results from the ACCENT II study	62.8
	The relationship between infliximab concentrations, antibodies to infliximab and disease activity in Crohn's disease [23]	95.5
	Infliximab therapy for Crohn's disease anoperineal lesions	70.3
	Discontinuation of infliximab therapy in patients with Crohn's disease in sustained complete remission (the STOP IT study): protocol for a double-blind, randomised, placebo-controlled, multicentre trial [24]	96.1
* Strikethrough: topics that remained excluded after the last Delphi round.		

In the final round, agreement percentages on topic inclusion are shown in the third column. Out of 20 topics, 15 were agreed upon in the first round. An additional 10 out of the remaining 15 were included in the second round. Three major reasons emerged from the panel’s explanations for topic inclusion: the most common reason was that the topic had been published within the last 10 years; another significant reason was that the topic closely aligned with the objectives for developing our (systematic review of the literature) SRL; and the final reason was that the topic addressed an area previously overlooked, filling a recognized gap in the research. Table 5 shows an in-depth description of the articles we decided to use.

**Table 5:** Data extraction RCT: randomized clinical trial; ADA: anti-drug antibody; CT-P13: biosimilar infliximab; IFX: infliximab; CD: Crohn’s disease; CDI: Clostridium difficile infection; TDM: therapeutic drug monitoring; RA: rheumatoid arthritis; UC: ulcerative colitis; IBD: inflammatory bowel disease; SC: subcutaneous; IV: intravenous; ATI:  antibodies to infliximab; TNFi: tumor necrosis factor-inhibiting

Author	Year of Publication	Study Design	Primary Research	Outcome Evaluation
Park et al. [13]	2023	RCT	Early serum IFX trough levels and ADA levels were compared in the CT-P13 (n = 100) and originator IFX (n = 98) groups.	Serum IFX trough levels at weeks 6 and 14 were excellent predictors of long-term clinical outcomes. There were no significant changes in serum IFX trough or ADA levels between CT-P13 and originator IFX.
Louis et al. [14]	2023	RCT	Adult CD patients in steroid-free clinical remission for more than 6 months who had been on combination treatment with IFX and immunosuppressant medication for at least 8 months were randomly allocated (1:1:1) to continue combination therapy, stop IFX, or cease immunosuppressive therapy.	Withdrawal of IFX should only be undertaken after a thorough evaluation of the risks and benefits for each patient, although discontinuing immunosuppressant medication may be a preferred option when contemplating therapeutic de-escalation.
Louie et al. [17]	2023	RCT	The research included 79 people aged 18 and up who were diagnosed with laboratory-confirmed CDI and had one or more past CDI episodes in the previous six months, as well as those with primary CDI who were at high risk of recurrence.	High-dose VE303 reduced recurrent CDI in people with laboratory-confirmed CDI with 1 or more past CDI episodes in the last 6 months, as well as those with initial CDI at high risk of recurrence, when compared to placebo. A bigger phase 3 investigation is required to validate these results.
Syversen et al. [18]	2021	RCT	The main objective was sustained illness management without disease worsening, as determined by disease-specific composite scores or patient-physician agreement on disease deteriorating, which resulted in a significant change in therapy.	In patients with immune-mediated inflammatory disorders receiving maintenance therapy with IFX, proactive TDM was more successful than treatment without TDM in maintaining disease control without deterioration.
Syversen et al. [19]	2021	RCT	411 people with RA, spondyloarthritis, psoriatic arthritis, UC, CD, or psoriasis started IFX treatment in 21 Norwegian institutions.	Over 30 weeks, proactive therapeutic drug monitoring did not substantially increase clinical remission rates in patients with immune-mediated inflammatory disorders starting IFX treatment compared to conventional therapy.
Schreiber et al. [20]	2021	RCT	This trial investigated the pharmacokinetics, symptomatic and endoscopic effectiveness, safety, and immunogenicity of a subcutaneous version of the IFX biosimilar CT-P13 SC to intravenous CT-P13 IV in patients with IBD.	The pharmacokinetic noninferiority of CT-P13 SC to CT-P13 IV, as well as the equivalent effectiveness, safety, and immunogenicity profiles, suggest CTP13 SC's potential applicability as an IBD therapy.
Jørgensen et al. [21]	2020	RCT	The 52-week, randomized, non-inferiority, double-blind, multicenter, phase 4 NOR-SWITCH research was followed by a 26-week open extension trial in which all patients were treated with CT-P13.	These exploratory subgroup analyses demonstrate that there are no substantial risks associated with switching from originator IFX to CT-P13 in CD and UC.
D'Haens et al. [22]	2018	RCT	From July 2012 to September 2015, 122 biologic-naïve adult patients with active CD were treated with IFX and an immunosuppressant in a double-blind study across 27 European locations.	They discovered that raising the dosage of IFX based on a combination of symptoms, biomarkers, and blood drug concentrations did not result in a higher percentage of patients experiencing corticosteroid-free clinical remission than increasing the dose just based on symptoms.
Vande Casteeleet al. [23]	2015	RCT	According to an examination of 1487 samples, 77.1% of patients had measurable and 22.9% had undetectable IFX concentrations, with 9.5% and 71.8%, respectively, testing positive for ATI.	The formation of ATI raises the likelihood of active illness, even at low concentrations and in the presence of a therapeutic dose of the medication during IFX maintenance treatment.
Buhl et al. [24]	2014	RCT	Patients with luminal Crohn’s disease who have been treated with IFX for at least 1 year and are in sustained complete clinical, biochemical, and endoscopic remission.	The project will provide new knowledge regarding how to optimally handle patients with CD in sustained remission on a TNFi and will help develop new therapeutic strategies for this patient group.

**Figure 2:**
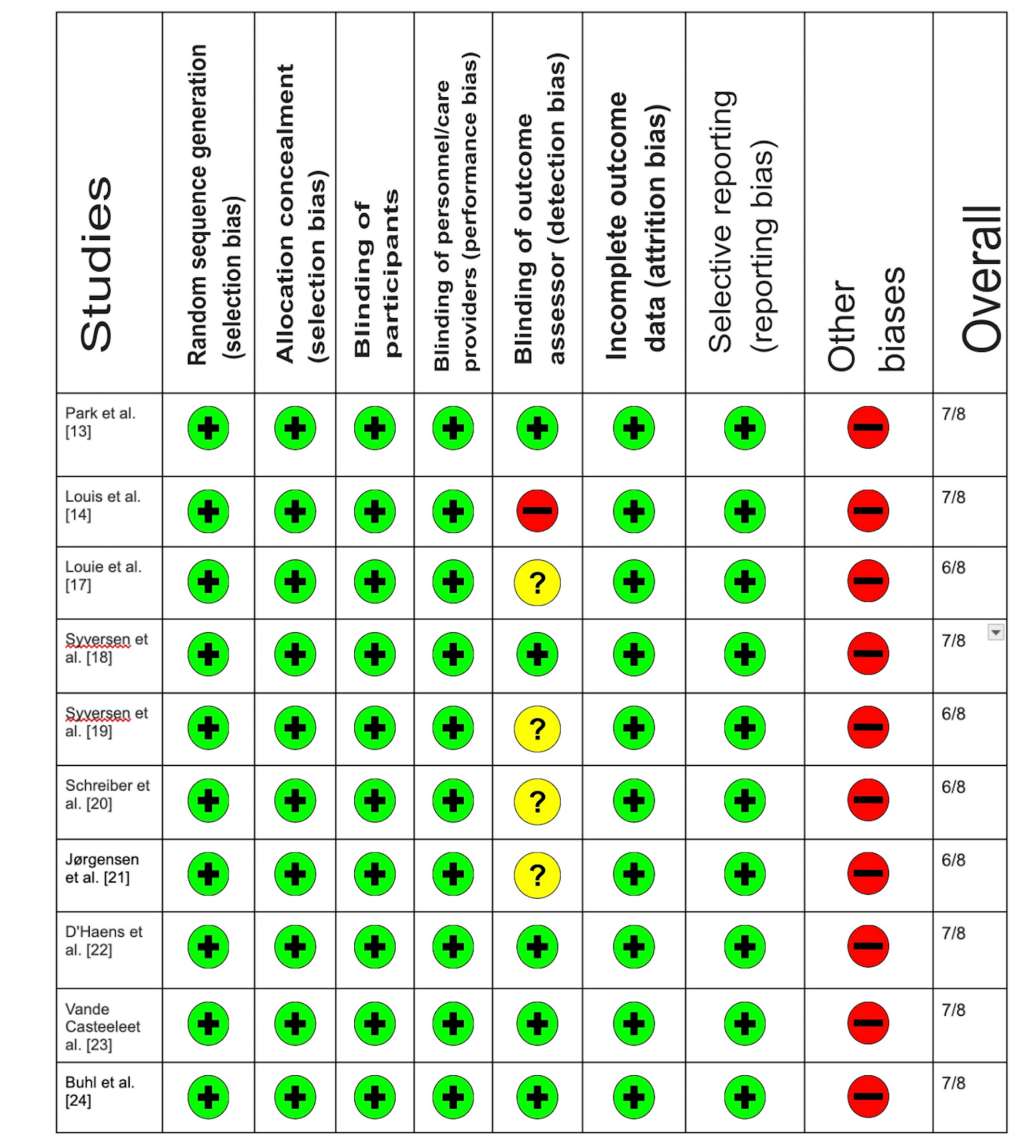
Cochrane risk of bias tool

Figure 2 presents the Cochrane risk of bias tool for randomized controlled trials.

Discussion

Through a thorough examination of the existing literature, we have gathered a variety of studies that evaluate the effectiveness of infliximab (IFX) in the treatment of inflammatory bowel disease (IBD). Park et al. conducted a study that found patients with biologic-naive Crohn's disease to have comparable levels of serum infliximab trough and anti-drug antibodies. Regardless of whether they received treatment with the biosimilar infliximab (CT-P13) or the original formulation, this observation held true [13]. A study [13] shows that measuring the lowest levels of infliximab in the blood at weeks six and 14 can tell us a lot about how likely it is that we will see clinical and endoscopic remission at weeks 30 and 54. Incorporating trough levels with markers of inflammation, such as fecal calprotectin or C-reactive protein (CRP), greatly improved the accuracy of predicting long-term remission outcomes [13].

The SPARE trial, as reported by Louis et al., found that patients who stopped infliximab while on combination therapy had a higher risk of relapse compared to those who either continued on the combination or switched to infliximab monotherapy [14]. This study examined patient characteristics that can help predict a higher likelihood of relapse and treatment failure. Interestingly, a significant number of patients who experienced a relapse showed a rapid response to infliximab retreatment without significantly affecting the overall duration of remission over a span of two years [14]. It is important to consider various factors, such as patient preferences, clinical profiles, and broader health policy and economic contexts, when making decisions about treatment scaling down [14].

Louie et al. talked about the results of a study that looked into how well VE303, a specific mix of eight Clostridia strains, stopped Clostridium difficile infection (CDI) [17]. A double-blind, placebo-controlled randomized controlled trial (RCT) conducted the study. This groundbreaking study showcased the effectiveness of VE303, making it the first documented case of a specific bacterial consortium proving its therapeutic benefits in a clinical setting [17]. During the eight-week primary efficacy period, the use of high-dose VE303 proved to be highly effective in preventing CDI recurrence in high-risk individuals. It is worth noting that all recurrences happened within the first 11 days, indicating swift colonization by VE303 strains and the restoration of gut microbiota [17]. The observed effect showed long-lasting results, as the majority of participants continued to experience the benefits up to week 24 [17].

Syversen et al. emphasized the advantages of proactive therapeutic drug monitoring (TDM) in patients with immune-mediated inflammatory diseases who are on maintenance therapy with infliximab. They found that this approach helps to maintain disease control more effectively compared to treatment without TDM [18,19]. However, another study by the same group found that initiating infliximab treatment did not significantly impact clinical remission rates with proactive TDM. This suggests that routine TDM might not be essential in the early stages of treatment for improving remission rates [18,19].

Schreiber et al.'s RCT demonstrated that subcutaneous (SC) CT-P13 is just as effective as the intravenous (IV) form in treating active IBD [20]. Whether starting with a post-dose loading or switching from IV at week 30, the SC formulation consistently maintained infliximab trough levels above the therapeutic threshold throughout the study [20]. The efficacy and safety profiles remained consistent for patients who continued subcutaneous (SC) treatment after receiving an intravenous (IV) dose loading, as well as for those who switched from IV to SC at week 30. This supports the use of the SC formulation as a viable and effective option for treating inflammatory bowel disease (IBD) [20].

In both the CD and ulcerative colitis (UC) groups, Jörgensen et al. evaluated the NOR-SWITCH trials and found no significant differences in effectiveness, safety, or immunogenicity between patients who received IFX and those who received CT-P13 [21]. These findings provide support for the clinical suggestion of transitioning from the original to the biosimilar infliximab in patients with IBD [21].

D'Haens et al. found no advantage in adjusting the dose of IFX for active CD based on a combination of symptoms, biomarkers, and serum concentrations [22]. This result highlights the difficulties of carrying out pure TDM trials, especially when biologic dose escalation is a common practice in clinical settings [22]. Meanwhile, Vande Casteele and colleagues discovered that certain levels of IFX and antibodies to infliximab (ATI) are associated with CD remission. This emphasizes the importance of conducting more research on how ATI affects the effectiveness of IFX and finding ways to minimize its development [23]. This extensive body of research highlights the importance of continuous investigations and practical applications in improving IFX treatment strategies for IBD [23].

Limitations

Genetic diversity and environmental factors: These factors can influence the response to IFX. Studies may not always account for these factors, potentially affecting the generalizability of the findings.

Patient adherence and real-world effectiveness: Clinical trial settings often have higher patient adherence rates than real-world settings. The effectiveness of IFX in routine clinical practice may differ from that observed in controlled trial environments.

Reporting of adverse effects: Inconsistent reporting of adverse effects and side effects across studies can lead to an incomplete understanding of the safety profile of infliximab.

## Conclusions

In conclusion, our systematic review of current literature highlights the nuanced role of IFX in treating IBD. Studies consistently show that serum infliximab trough levels, along with markers of inflammation, are predictive of long-term remission, providing valuable insights for clinical decision-making. The SPARE trial underscores the risks of discontinuing IFX in patients on combination therapy, emphasizing the importance of individualized treatment plans. The innovative use of VE303, a bacterial consortium, marks a significant advancement in preventing Clostridium difficile infection, showcasing the potential of microbiota-targeted therapies. While proactive TDM shows benefits in maintenance therapy, its role during the induction phase remains debatable. The non-inferiority of subcutaneous CT-P13 compared to intravenous formulations offers flexibility in treatment administration without compromising efficacy or safety. The NOR-SWITCH trials further validate the interchangeability of original and biosimilar IFX formulations. Despite these advancements, the challenge of optimizing IFX dosing strategies persists, particularly in the context of anti-drug antibodies. Continued research is essential to refine these strategies and enhance patient outcomes in IBD management.

## References

[1] Miranda EF, Nones RB, Kotze PG (2021). Correlation of serum levels of anti-tumor necrosis factor agents with perianal fistula healing in Crohn's disease: a narrative review. Intest Res.

[2] Argollo M, Kotze PG, Kakkadasam P, D'Haens G (2020). Optimizing biologic therapy in IBD: how essential is therapeutic drug monitoring?. Nat Rev Gastroenterol Hepatol.

[3] Ye BD, Pesegova M, Alexeeva O (2019). Efficacy and safety of biosimilar CT-P13 compared with originator infliximab in patients with active Crohn’s disease: an international, randomised, double-blind, phase 3 non-inferiority study. Lancet Lond Engl.

[4] Strik AS, van de Vrie W, Bloemsaat-Minekus JPJ (2018). Serum concentrations after switching from originator infliximab to the biosimilar CT-P13 in patients with quiescent inflammatory bowel disease (SECURE): an open-label, multicentre, phase 4 non-inferiority trial. Lancet Gastroenterol Hepatol.

[5] Fiorino G, Ruiz-Argüello MB, Maguregui A (2018). Full interchangeability in regard to immunogenicity between the infliximab reference biologic and biosimilars 
CT-P13 and SB2 in inflammatory bowel disease. Inflamm Bowel Dis.

[6] Gonczi L, Vegh Z, Golovics PA (2017). Prediction of short- and medium-term efficacy of biosimilar infliximab therapy. do trough levels and antidrug antibody levels or clinical and biochemical markers play the more important role?. J Crohns Colitis.

[7] Feuerstein JD, Nguyen GC, Kupfer SS, Falck-Ytter Y, Singh S (2017). American gastroenterological association institute clinical guidelines committee. American gastroenterological association institute guideline on therapeutic drug monitoring in inflammatory bowel disease. Gastroenterology.

[8] Farkas K, Rutka M, Golovics PA (2016). Efficacy of infliximab biosimilar CT-P13 induction therapy on mucosal healing in ulcerative colitis. J Crohns Colitis.

[9] Ben-Horin S, Yavzori M, Benhar I (2016). Cross-immunogenicity: antibodies to infliximab in Remicade-treated patients with IBD similarly recognise the biosimilar Remsima. Gut.

[10] Paul S, Del Tedesco E, Marotte H (2013). Therapeutic drug monitoring of infliximab and mucosal healing in inflammatory bowel disease: a prospective study. Inflamm Bowel Dis.

[11] Sands BE, Anderson FH, Bernstein CN (2004). Infliximab maintenance therapy for fistulizing Crohn's disease. N Engl J Med.

[12] Hanauer SB, Feagan BG, Lichtenstein GR (2002). Maintenance infliximab for Crohn’s disease: the ACCENT I randomised trial. Lancet Lond Engl.

[13] Park J, Cheon JH, Lee KM (2023). Early infliximab trough levels predict the long-term efficacy of infliximab in a randomized controlled trial in patients with active crohn’s disease comparing, between ct-p13 and originator infliximab. Gut Liver.

[14] Louis E, Resche-Rigon M, Laharie D (2023). Withdrawal of infliximab or concomitant immunosuppressant therapy in patients with Crohn's disease on combination therapy (SPARE): a multicentre, open-label, randomised controlled trial. Lancet Gastroenterol Hepatol.

[15] Page MJ, McKenzie JE, Bossuyt PM (2021). The PRISMA 2020 statement: an updated guideline for reporting systematic reviews. BMJ.

[16] Wu SS, Sun F, Zhan SY (2017). Bias risk assessment: (3) Revised Cochrane bias risk assessment tool for individual randomized, cross-over trials (RoB2.0) (Article in Chinese). Zhonghua Liu Xing Bing Xue Za Zhi.

[17] Louie T, Golan Y, Khanna S (2023). VE303, a defined bacterial consortium, for prevention of recurrent Clostridioides difficile infection: A randomized clinical trial. JAMA.

[18] Syversen SW, Jørgensen KK, Goll GL (2021). Effect of therapeutic drug monitoring vs standard therapy during maintenance infliximab therapy on disease control in patients with immune-mediated inflammatory diseases: a randomized clinical trial. JAMA.

[19] Syversen SW, Goll GL, Jørgensen KK (2021). Effect of therapeutic drug monitoring vs standard therapy during infliximab induction on disease remission in patients with chronic immune-mediated inflammatory diseases: a randomized clinical trial. JAMA.

[20] Schreiber S, Ben-Horin S, Leszczyszyn J (2021). Randomized controlled trial: subcutaneous vs intravenous infliximab CT-P13 maintenance in inflammatory bowel disease. Gastroenterology.

[21] Jørgensen KK, Goll GL, Sexton J (2020). Efficacy and safety of CT-P13 in inflammatory bowel disease after switching from originator infliximab: exploratory analyses from the NOR-SWITCH main and extension trials. BioDrugs.

[22] D'Haens G, Vermeire S, Lambrecht G (2018). Increasing infliximab dose based on symptoms, biomarkers, and serum drug concentrations does not increase clinical, endoscopic, and corticosteroid-free remission in patients with active luminal crohn’s disease. Gastroenterology.

[23] Vande Casteele N, Khanna R, Levesque BG (2015). The relationship between infliximab concentrations, antibodies to infliximab and disease activity in Crohn's disease. Gut.

[24] Buhl SS, Steenholdt C, Brynskov J, Thomsen OØ, Bendtzen K, Ainsworth MA (2014). Discontinuation of infliximab therapy in patients with Crohn's disease in sustained complete remission (the STOP IT study): protocol for a double-blind, randomised, placebo-controlled, multicentre trial. BMJ Open.

